# Randomized Controlled Trial to Compare the Safety and Efficacy of Tamsulosin, Solifenacin, and Combination of Both in Treatment of Double-J Stent-Related Lower Urinary Symptoms

**DOI:** 10.1155/2013/752382

**Published:** 2013-10-23

**Authors:** Essam Shalaby, Abul-fotouh Ahmed, Aref Maarouf, Iman Yahia, Mohamed Ali, Ammar Ghobish

**Affiliations:** ^1^Department of Urology, Suez Canal University, P.O. Box 224, Ismailia 41522, Egypt; ^2^Department of Urology, King Khalid Hospital, P.O. Box 1985, Al-Kharj 11942, Saudi Arabia; ^3^Department of Urology, Al-Azhar University, Nasr City, Cairo 11884, Egypt; ^4^Department of Urology, Salman Bin Abdul-Aziz University, P.O. Box 173, Al-Kharj 11942, Saudi Arabia; ^5^Department of Urology, Faculty of Medicine, Zagazig University, P.O. Box 203, Zagazig 44519, Egypt; ^6^Department of Urology, King Abdallah Hospital, P.O. Box 60, Bisha 61922, Saudi Arabia; ^7^Department of Urology, Insurance Hospital, Ismailia 41528, Egypt

## Abstract

*Purpose*. We evaluated the effectiveness and safety of tamsulosin, solifenacin, and combination of both in reducing double-J stent-related lower urinary symptoms. *Materials and Methods*. A total of 338 patients with double-J ureteral stenting were randomly divided, postoperatively, into 4 groups. In group I (*n* = 84), no treatment was given (control group), group II (*n* = 85) received tamsulosin 0.4 mg daily, group III (*n* = 84) received solifenacin 10 mg daily, and group IV (*n* = 85) received a combination of both medications. Before insertion and 2 weeks after, all patients completed the International Prostate Symptom Score (IPSS), quality of life component of the IPSS (IPSS/Qol), Overactive Bladder Questionnaire (OAB-q), and Visual Analogue Pain Scale (VAPS) questionnaire. *Results*. The demographics and preoperative questionnaires scores of all groups were comparable. There were statistically significant differences in all scores in favour of groups II, III, and IV as compared to control group (*P* value < 0.005). Group IV showed statistically significant differences in total IPSS, QoL score, and OAB-q score as compared to groups II and III (*P* value < 0.001). *Conclusions*. Combined therapy of tamsulosin and solifenacin significantly alleviated lower urinary symptoms associated with double-J stents as compared to either medication alone.

## 1. Introduction

The double-J stents are common tools and integral part used in endourologic practices. Double-J stents play a major role in a wide range of situations to prevent or to relieve ureteral obstruction [1, 2]. Despite the usefulness of double-J stent, some of the patients might encounter stent-related morbidities such as urinary tract infection (UTI), lower urinary tract symptoms (LUTS), stent-related body pain, and hematuria. These symptoms represent a prevalent problem with considerable effects on the quality of life, substantial general health, work performance, and sexual matters in both genders [3–5]. The pathophysiology of stent-related symptoms remains unclear. However, the pain and LUTS caused by stent placement has been attributed to lower ureter and bladder spasm due to local irritation of the stent [6]. Some previous studies indicated oral agents such as alpha-1-blockers and antimuscarinic agents to relieve LUTS associated with double-J ureteral stents [7–10]. 

Tamsulosin acts as a selective inhibitor of *α*-1a/1d-mediated contraction of the smooth muscles in distal ureter, bladder trigone, and bladder neck [11]. It is thought that relaxing these smooth muscles decreases bladder outlet resistance and voiding pressure, with beneficial effect on stent related LUTS [7, 10]. Solifenacin acts as a muscarinic receptor antagonist used for treatment of patients with overactive bladder (OAB) [12, 13] and might be effective as well for stent-related symptoms [8–10]. 

In this prospective study we evaluated the effectiveness and safety of alpha-1-blocker (tamsulosin), antimuscarinic (solifenacin), and combination of both medications in reducing double-J stent-related LUTS, using the International Prostate Symptom Score (IPSSs), quality of life component of the IPSS (IPSS/Qol), Overactive Bladder Questionnaire (OAB-q), and Visual Analogue Pain Scale (VAPS) questionnaire.

## 2. Patients and Methods

### 2.1. Study Population and Design

This multicentre, prospective, randomized, and comparative study was carried out between November 2009 and March 2013. Patients were selected among those underwent retrograde double-J ureteral stent placement, before ESWL or following ureterorenoscopy (URS), ureterolithotomy, percutaneous nephrolithotomy (PCNL), and endoscopic endopyelotomy. The study was carried out by five urologists at four institutions (King Khalid Hospitals “KKH,” Salman Bin Abdul-Aziz University Hospital “SAUH” (Al-Kharj, KSA), King Abdullah Hospital “KAH” (Bisha, KSA), and Ismailia Insurance Hospital “IIH” (Ismailia, Egypt)). Patients ≥18 years of age with unilateral double-J ureteral stent who agreed to random allocation of treatment were eligible for enrolment. Patients were excluded if they met any of the following criteria: (1) age less than 18 years, (2) pregnant women, (3) history of previous ureteral stenting, (4) bilateral stents, (5) long-term stenting (on regular exchange), (6) bladder pathology, (7) benign prostatic hyperplasia, (8) overactive bladder, (9) urinary tract infection, or (9) previous use of selective alpha-1-blocker and/or antimuscarinic agent.

### 2.2. Study Procedure

A total of 338 patients were enrolled in study (172 patients from KKH and SAUH, 102 patients from KAH, and 64 patients from IIH). A computer randomization program (http://www.randomization.com/) was used to allocate patients into four groups. We used randomization in blocks of four, and each center had its own list to keep the groups closely balanced. Patients in group I (*n* = 84) received no treatment (control group) (C group), patients in group II (*n* = 85) received a daily oral dose of tamsulosin 0.4 mg (T group), patients in group III (*n* = 84) received solifenacin succinate 10 mg (S group), and patients in group IV (*n* = 85) received a combination of the two medications once daily (T/S group). Routine preoperative evaluation was done for the planned operative procedures. A polyurethane ureteral stents were used in all patients. The length and calibre were adjusted for each patient. Only the coiled distal end was present in the bladder without any part of distal shaft. Routinely, X-ray abdominal film was done for all patients before home discharge to confirm the proper stent positioning. The protocol and all study procedures were conducted in conformity with the ethical guidelines of the Declaration of Helsinki of 1975. The study protocol was approved by the ethics committee of the participating hospitals, and all patients enrolled in this study provided written informed consent.

### 2.3. Patients Assessment and Outcome Measurements

Before and two weeks after stent insertion, all patients completed written International Prostate Symptom Score/quality of life component of IPSS (IPSS/QoL), Overactive Bladder Questionnaire (OAB-q), and Visual Analogue Pain Scale (VAPS) questionnaires. The IPSS was divided into the total score, voiding symptom score, and storage symptom score, and each one was compared. Visual Analogue Pain Scale graded from 1 (minimal or no symptoms) to 10 (symptoms of maximal severity). 

### 2.4. Statistical Analysis

Using SPSS software ver. 18.0 (SPSS Inc., Chicago, IL, USA), statistical analysis was performed. Chi-square test, ANOVA, and one-way repeated-measures ANOVA were used for comparisons between the 4 groups. When comparing postinsertion to preinsertion scores, a statistically significant difference reflects poor control of stent related symptoms. The power used was 0.80, and the level of significance was 5%.

## 3. Results

A total of 338 patients were enrolled in the study, and 327 patients completed the study (3 patients from group I, 3 patients from group II, 4 patients from group III, and 1 patient from group IV dropped out). Group I (81 patients) consisted of 50 men and 31 women (mean age: 44.0 ± 15.2 years), group II (82 patients) consisted of 55 men and 27 women (mean age: 41.3 ± 17.1 years), group III (80 patients) consisted of 57 men and 23 women (mean age: 40.5 ± 18.6 years), and group IV (84 patients) consisted of 58 men and 26 women (mean age: 43.6 ± 17.6 years). The main indication of ureteral double-J stent placement was URS and ureterolithotomy. All patients completed the necessary questionnaires. There were no statistically significant differences between groups regarding patient's demographics, treatment indications, and preoperative questionnaires scores (Tables 1 and 2). 

Therapies were well tolerated, and no patients discontinued the treatments because of side effects. When comparing the pre- and postinsertion scores, we found that there was a statistically significant difference in all evaluated scores in the control group, and this represents poor control of stent related symptoms. In single therapy groups (T and S groups) each drug controlled voiding symptoms (*P* = 0.698 and 0.411) but not other stent related symptoms. However, in combination therapy (T/S group) the differences were nonsignificant in all evaluated scores indicating maximum symptom control.

 When comparing poststenting scores among different groups, there were a statistically significant differences in all scores in favour of groups II, III, and IV as compared to group I (*P* value < 0.005). However when we compared each group by one-way ANOVA at each time point separately, there was no statistically significant difference between groups II and III as regards (total, storage, and voiding) IPSS scores (*P* = 0.352, 0.07, and 0.513, resp.), although there was statistically significant difference in OAB-q and VAPS scores in favour of group II (*P* = 0.00) (Table 3). Group IV patients who received combination therapy showed a statistically significant difference in all scores as compared to monotherapy (II, III) groups (*P* value < 0.001). This confirmed the superiority of combination therapy in overcoming stent-related symptoms as compared to monotherapy. 

## 4. Discussion

The results of this prospective, randomized, controlled trial showed that, the combined use of tamsulosin and solifenacin improved the QoL and alleviated LUTS associated with double-J ureteral stents, better than either drug alone and well tolerated.

Stent discomfort is believed to affect over 80% of patients [14, 15]. Patients with indwelling stents have been known to complain of a variety of stent-related symptoms, typically: storage, voiding, OAB symptoms, haematuria, and pain. These symptoms are believed to be unavoidable and associated with reduced health-related quality of life [4].

The exact pathophysiology of stent related symptoms remains unknown; it could be related to lower ureteral smooth muscle spasms and local irritation to neuronal-rich trigonal mucosa, which contains *α*-1D receptors and bladder instability which give symptoms similar to benign prostatic hyperplasia [6, 16, 17]. To overcome the bothering short term stent related symptoms, some investigators reported that stent length, girth adjustment and avoiding distal end crossing the midline are essential and significantly decrease stent's symptoms [18]. A different design was introduced in the Tail Stent model (Microvasive Urology/Boston Scientific) with proximal 7F pigtail and tapered end 3F tail that lie in the bladder. This stent was compared to standard 7F double-J stents in a randomized single-blind trial involving 60 patients and showed significant reduction in the stent-related symptoms [19, 20]. This was in contrary to that reported by Hao et al. [21] and Thomas [6] whom showed no significant effect of length and girth on stent symptoms. Damiano et al. [22] reported that there was no symptoms difference between stent with different size, whereas there was a tendency of small diameter stents to dislodge more often. Chew et al. [23] reported that changing in body position led to movement of distal end within the bladder and induced more trigonal irritation and stent related symptoms.

Lang and associates [24] stated that a possible mechanism of relief of stent-related symptoms could be smooth muscle relaxation of lower ureter and trigone as well as reducing ureteric motility. Wang and his colleagues [25] suggested that relaxation of bladder neck/prostatic smooth muscle, with consequent reduction in voiding pressure and urinary reflux, is other possible mechanisms for control of stent-related symptoms, setting a rationale behind using alpha blockers in overcoming ureteral stent symptoms.

Another mechanism was thought to be related to stent itself which may unmask or exacerbate preexisting subclinical detrusor overactivity causing involuntary bladder contraction, which induces overactive bladder symptoms, setting a rationale behind using antimuscarinic agents to improve stent-related symptoms [26–28]. The symptoms of incontinence and urge incontinence may be explained by stent migration into the proximal urethra which interferes with the urethral sphincter mechanism of continence [28]. 

The effectiveness of different therapeutic protocols aiming to improve ureteral stent-related symptoms is under investigation. In the present study, we found that both tamsulosin and solifenacin monotherapy poorly controlled ureteral stent related symptoms (*P* value 0.000). The effectiveness of alpha blockers in controlling double-J stent-related symptoms was reported previously. Wang et al. [7] in a prospective randomized study comparing tamsulosin to placebo in 79 patients using (USSQ) reported that tamsulosin improved stent related urinary symptoms, QoL, and they recommended its routine use. Also Damiano et al. [17] reported that administration of tamsulosin has a positive effect on stent-related urinary symptoms, QoL, and VAPS, although this study was not double-blinded or placebo-controlled. Also, several studies reported that other alpha-blocker alfuzosin improved stent-related symptoms and quality of life and reduced analgesic demand compared to the placebo group [29, 30]. However, to our knowledge, no studies compared the effectiveness of different alpha blockers or antimuscrinic agents on stent-related symptoms. Kuyumcuoglu et al. [31] reported in a prospective randomized study that tamsulosin was not different than placebo in controlling stent-related symptoms.

In our study, solifenacin monotherapy poorly controlled stent-related symptoms evidenced by statistically significant differences in the IPSS total score, storage subscore, QoL, OAB-q, and VAP scores pre- and post-stent insertion. When compared to placebo almost all scores showed statistically significant differences except for storage subscore which indicated that solifenacin was better than placebo, in controlling irritative LUTS. Similarly, Lee et al. [9] reported in a prospective, randomized, and placebo-controlled study that postoperative solifenacin use was effective and well tolerated for the treatment of LUTS, stent-related body pain, and hematuria irrespective of gender in patients undergoing ureteroscopic lithotripsy (URSL) and double-J stent indwelling. 

Also, researchers studied the effect of others antimuscrinic agents in reducing the negative symptoms associated with double-J ureteral stent. Norris et al. [32] reported in a prospective, randomized, and double-blinded placebo-controlled study that there were no differences between oxybutynin and placebo in controlling stent-related symptoms. However, they recommended further study on a large number of patients for optimal management of ureteral stent symptoms. Kuyumcuoglu et al. [31] reported that tolterodine SR 4 mg was not different than anti-inflammatory and alpha blocker in controlling stent-related symptoms. In contrast to this data Park et al. [33] in a prospective randomized controlled study reported that tolterodine was significantly able to improve pain and urinary symptom index scores when compared with alfuzosin and placebo. 

A limitation of our study was the lack of patient homogeneity (as we included patients with different urologic procedures). However, the indications of double-J stent insertion were statistically similar in the four groups, and our main focus was to compare the efficacy of tamsulosin versus solifenacin versus combined treatment as this has been studied in only few literatures. Our findings showed that combined therapy was better than either tamsulosin or solifenacin monotherapy in reducing stent related symptoms. The superiority of combined tamsulosin and solifenacin therapy was also reported previously by Lim and his colleagues [34] who reported in nonrandomized, retrospective study that combined use of solifenacin and tamsulosin was significantly better than either drug alone in reducing stent related symptoms. However, the small groups of each scale made it difficult to verify the statistical significance, and authors recommended further large-scale, randomized, and prospective study to get more accurate information. In contrast, Lee et al. [35] in their prospective randomized study over 20 patients using a combination of Tamsulosin and tolterodine reported no statistically significant difference when compared to placebo, and also when combination therapy was compared to tamsulosin monotherapy, no beneficial effect was reported. They stated that correct stent positioning and verification of its location were more important than medication for lessening stent-related storage symptoms. 

## 5. Conclusions

Combination therapy of tamsulosin and solifenacin is significantly better than either drug alone in reducing LUTS associated with double-J stents. Combination therapy should be strongly considered for patients who complain of stent-related symptoms. However, in our opinion, there is need for further studies to compare the effectiveness of combination of different alpha blockers and antimuscrinic agents in order to optimize medical therapy for treatment of symptoms related to stent placement.

## Figures and Tables

**Table 1:** Basic characteristics of studied patients.

Variables	Group I	Group II	Group III	Group IV	*P* value
	C group	T group	S group	T/S group	
Number of patients	81	82	80	84	< 0.05
Mean age (years)	44.0 ± 15.2	41.3 ± 17.1	40.5 ± 18.6	43.6 ± 17.6	
Sex (male : female)	1.6 : 1	2 : 1	2.5 : 1	2.2 : 1	
Indications of stent placement					
URS/ureterolithotomy	56	57	59	63	
PCNL	07	10	07	05	
ESWL	16	14	12	13	
Endopyelotomy	02	01	02	03	

URS: Ureterorenoscopy; PCNL: Percutaneous Nephrolithotomy; ESWL: Extracorporeal Shock Wave Lithotripsy.

**Table 2:** Comparisons of IPSS/QoL, VAP scale, and OAB-q scores in all groups.

	Group I	Group II	Group III	Group IV	*P* value
	C group	T group	S group	T/S group	
IPSS (storage symptom score)					
Preinsertion	4.18 ± 2.48	4.25 ± 2.54	4.30 ± 2.65	4.30 ± 2.41	0.989
2 weeks after insertion	8.24 ± 3.44	7.68 ± 3.66	6.62 ± 3.92	4.66 ± 3.24	0.000
IPSS (voiding symptom score)					
Preinsertion	4.62 ± 2.86	4.66 ± 2.66	4.75 ± 2.55	4.62 ± 2.46	0.988
2 weeks after insertion	7.22 ± 2.46	4.82 ± 2.62	5.10 ± 2.82	3.82 ± 2.02	0.000
IPSS (total score)					
Preinsertion	8.80 ± 4.42	8.91 ± 4.26	9.05 ± 3.87	8.92 ± 4.12	0.986
2 weeks after insertion	15.46 ± 4.28	12.40 ± 4.50	11.72 ± 4.77	8.48 ± 4.22	0.000
Qol score					
Preinsertion	1.82 ± 1.62	1.78 ± 1.70	1.62 ± 1.54	1.77 ± 1.44	0.862
2 weeks after insertion	4.12 ± 1.76	2.80 ± 1.52	3.36 ± 1.77	1.88 ± 1.22	0.000
OAB-q score					
Preinsertion	8.2 ± 1.2	8.3 ± 1.3	8.1 ± 1.5	8.5 ± 1.3	0.255
2 weeks after insertion	20.53 ± 2.22	14.93 ± 1.62	12.42 ± 1.42	8.44 ± 1.22	0.000
VAPS score					
Preinsertion	2.30 ± 1.46	2.55 ± 1.84	2.69 ± 1.46	2.22 ± 1.27	0.172
2 weeks after insertion	5.88 ± 1.65	3.23 ± 1.23	4.36 ± 1.44	2.69 ± 1.41	0.000

IPSS: International Prostate Symptom Score; QoL: quality of life; OAB-q: Overactive Bladder Questionnaire; VAPS: Visual Analogue Pain Scale.

**Table 3:** Comparison between group II and III two weeks post insertion of Double-J stent.

	Group II	Group III	*P* value
	T group	S group	
IPSS Storage symptom score	7.68 ± 3.66	6.62 ± 3.92	0.07
IPSS Voiding symptom score	4.82 ± 2.62	5.10 ± 2.82	0.513
IPSS Total score	12.40 ± 4.5	11.72 ± 4.77	0.352
QoL score	2.80 ± 1.52	3.36 ± 1.77	0.013
OAB-q score	14.93 ± 1.62	12.42 ± 1.42	0.000
VAPS score	3.23 ± 1.23	4.36 ± 1.44	0.000

IPSS: International Prostate Symptom Score; QoL: Quality of Life; OAB-q: Overactive Bladder Questionnaire; VAPS: Visual Analogue Pain Scale.
